# Supplementary material for: Analysis of migration of pathogenic drug-resistant bacteria to soils and groundwater after fertilization with sewage sludge
Source: PLoS One. 2021 Dec 16;16(12):e0256936. doi: 10.1371/journal.pone.0256936 (PMC8675741; doi:10.1371/journal.pone.0256936)
Supplement: Attachment — Submitted filename: Response to Reviewers EN.docx [file pone.0256936.s001.docx]

**Response to Editors and Reviewers**

I would like to thank the Editors and both Reviewers very much for their time devoted to reviewing the manuscript. Thank you also very much for the valuable comments in the reviews.

**Journal Requirements:**

1. The required names of the submitted files were introduced according to the recommendations of the Editors and Reviewers.
2. The Methods section provides additional details on the experimental materials (soil and sewage sludge) that were used for the study.
3. The source of funding for the research and publication comes from the Ministry's funds for scientific research of the Częstochowa University of Technology and it is necessary to provide the information in Acknowledgments: „The scientific research was funded by the statute subvention of Czestochowa University of Technology, Faculty of Infrastructure and Environment”. Such an entry in the publication is necessary for us to receive a refund of the cost of the publication. It is required at the Częstochowa University of Technology. What should I do then? As recommended, I removed Acknowledgments. I am asking You to make the necessary changes on my behalf.
4. "The funders had no role in study design, data collection and analysis, decision to publish, or preparation of the manuscript."
5. Answer as in points 3 and 4
6. The manuscript includes the results of the research along with the dataset underlying the findings presented. The data on drug resistance in the tables are specific and therefore presented in this extended form. Therefore, a separate link to the data is not included. They are available in the manuscript. Based on the data contained in the publication, it is possible to reproduce and verify the entire research process.
7. I logged into Editorial Manager with my ORCID ID. I filled in the details.

**Responses to Reviewers**

**Reviewer 1**

Thank you for your time and valuable comments in your review.

Changes to the text are highlighted in yellow.

**Recommended revisions have been made to the Abstract, Introduction to the study, and Research Methodology.**

**Answer regarding Tables 3 and 4**: in the tables, the amounts of *Escherichia coli, Clostridium perfringens and Proteus vulgaris* bacteria are given in the form of so-called bacterial titer. The table incorrectly used the description "Bacteria count". The incorrect description has been corrected.

The microbial titer is the smallest volume of test material in which there is at least one living cell of the indicator microorganism. The titer determination is used to determine the degree of microbial contamination of the test material. The coliform titer test is most commonly performed because it is a bacterium that is found in human and animal feces. The *coliform*titer determination is the primary method for assessing whether water or another environment (soil) is contaminated with feces.The coliform titer is different from another indicator called the coliform index. The coliform index provides the number of coliform bacteria in 1 dm³. In determining the index, cultures made on Endo medium agar and colonies identified as coliform are counted.

The number of colony-forming bacteria units in the tables described as "Total bacteria count [CFU/1ml]" was determined for the total count of mesophilic bacteria,the *Enterococcus and Enterobacteriaceae* family*.*

An increased dose of sewage sludge applied to the soil fertilization caused an increase in the contamination with *Escherichia coli* of the fertilised soil and the water infiltrating it.

**Answer concerning Tables 5, 6, 7 and 8**: the results of drug resistance tests are related to various pathogenic groups of microorganisms from different environments tested. Tables 6 - 8 also show which groups of potentially pathogenic microorganisms were found after application of which doses of fertilizing sewage sludge. Data from Tables 5 -7 are compared in the description of the study results. The Reviewer's valuable comments on the form of presentation will be used in planning the next experiments.

**Reviewer 2**

Thank you very much for your valuable comments on our manuscript. I would also like to thank the Reviewer for appreciating the really large amount of organizational and research work.

Changes suggested and recommended by the Reviewer have been made to the paper.

Changes to the text are highlighted in green.

**Abstract line 13-23**

A brief characterization of the main results has been added in the Abstract.

**Materials and Methods line 99-100**

The methodology for sanitary analysis determination has been complemented

**Materials and Methods line 135**

Corrected as recommended

**Results Table 1. line 168-171**

The horizontal lines in Table 1 have been added.

**Results line 223-225**

This part has been complemented

**Results line 231-233**

The sentence has been removed

**Results Table 6. and Table 7. line 240 and 252**

The values presented in these tables represent sewage sludge application rates after which resistant bacteria occurred in the aquatic and soil environments.As suggested by the Reviewer, an additional explanatory description has been added below the table.

**Discussion line 317-320**

Thank you for finding the mistake. A previous value was incorrectly repeated. A correction has been made to the text.

**Discussion line 322**

A correction has been made to the text.

**Manuscript**

Units has been checked.
