# Supplementary material for: Analysis of migration of pathogenic drug-resistant bacteria to soils and groundwater after fertilization with sewage sludge
Source: PLoS One. 2021 Dec 16;16(12):e0256936. doi: 10.1371/journal.pone.0256936 (PMC8675741; doi:10.1371/journal.pone.0256936)
Supplement: Attachment — Submitted filename: Responses to Reviewer 2.docx [file pone.0256936.s002.docx]

**Responses to Reviewer 2**

Thank You very much for Your valuable comments on our work.

A correction has been made to the text.

Changes to the text are highlighted in green.
